# Supplementary material for: Dynamics Learning Rate Bias in Pigeons: Insights from Reinforcement Learning and Neural Correlates
Source: Animals (Basel). 2024 Feb 1;14(3):489. doi: 10.3390/ani14030489 (PMC10854969; doi:10.3390/ani14030489)
Supplement: Supplementary file 1 [file animals-14-00489-s001.zip › animals-2832459-supplementary.pdf]

Supplementary

P014

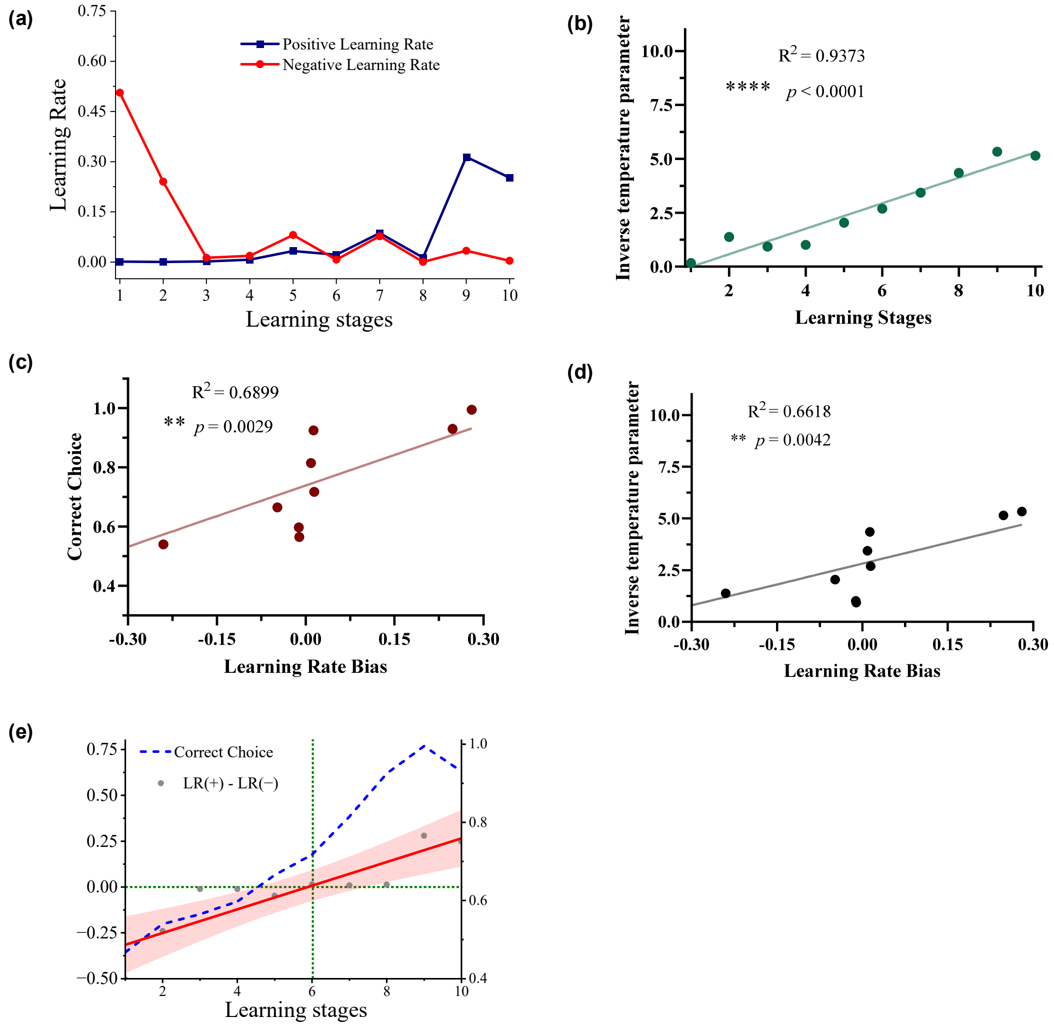

Figure S1. The model results of P014. (a) (a, b) Dynamic learning rate and inverse temperature parameter β of Model 2 (RLB) during the learning process. (c, d) Relationship between learning rate bias and choice performance, inverse temperature parameter β. (e) Dynamic learning rate (LR) bias and choice performance as a function of learning stages.

P093

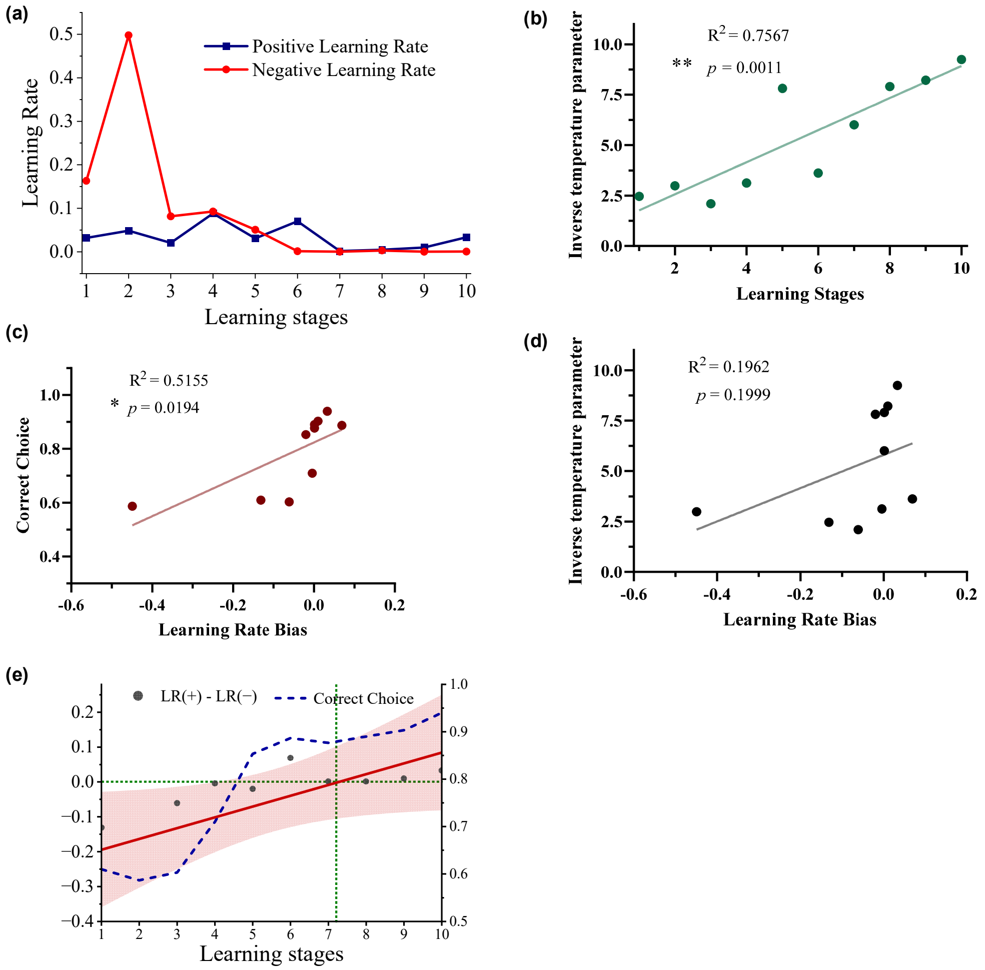

Figure S2. The model results of P093. (a) (a, b) Dynamic learning rate and inverse temperature parameter β of Model 2 (RLB) during the learning process. (c, d) Relationship between learning rate bias and choice performance, inverse temperature parameter β. (e) Dynamic learning rate (LR) bias and choice performance as a function of learning stages.

P021

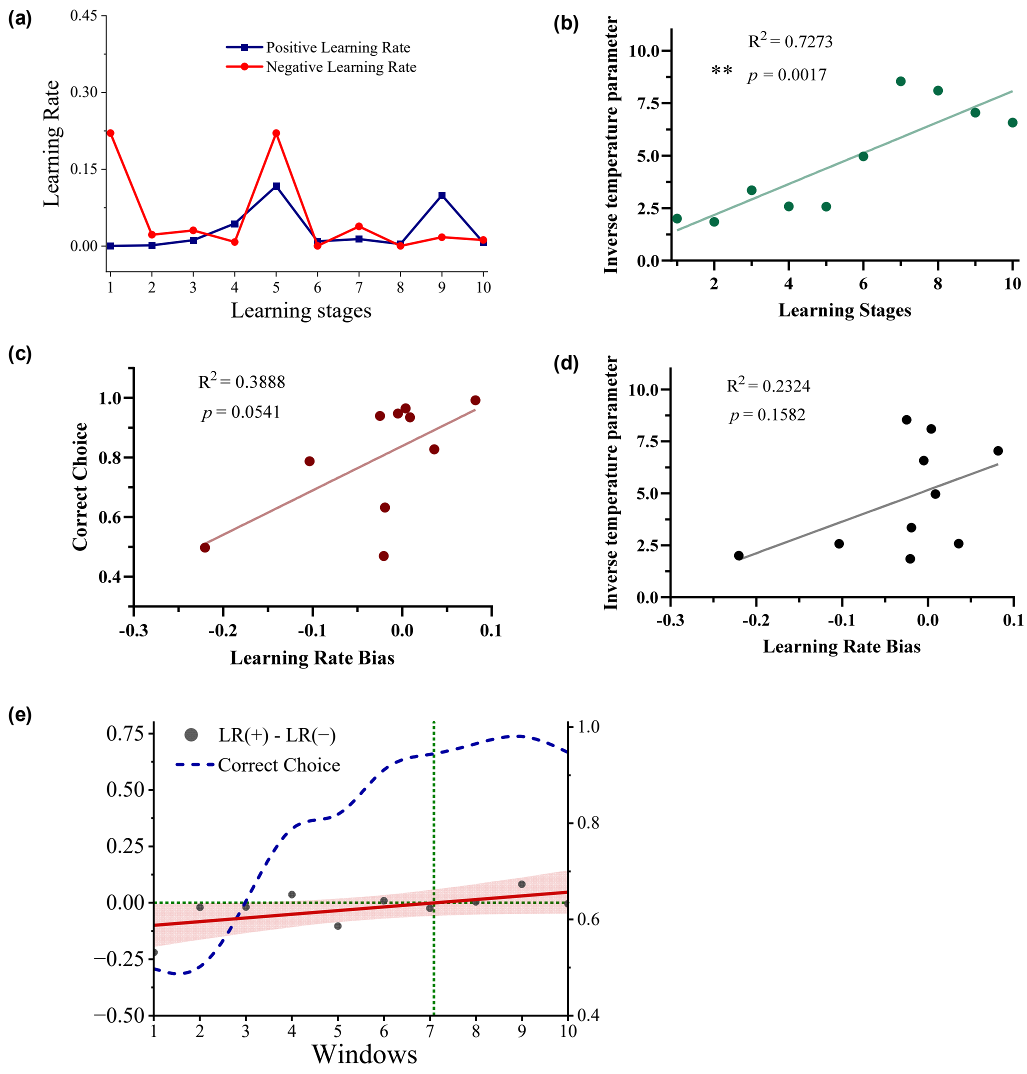

Figure S3. The model results of P021. (a) (a, b) Dynamic learning rate and inverse temperature parameter β of Model 2 (RLB) during the learning process. (c, d) Relationship between learning rate bias and choice performance, inverse temperature parameter β. (e) Dynamic learning rate (LR) bias and choice performance as a function of learning stages.

P029

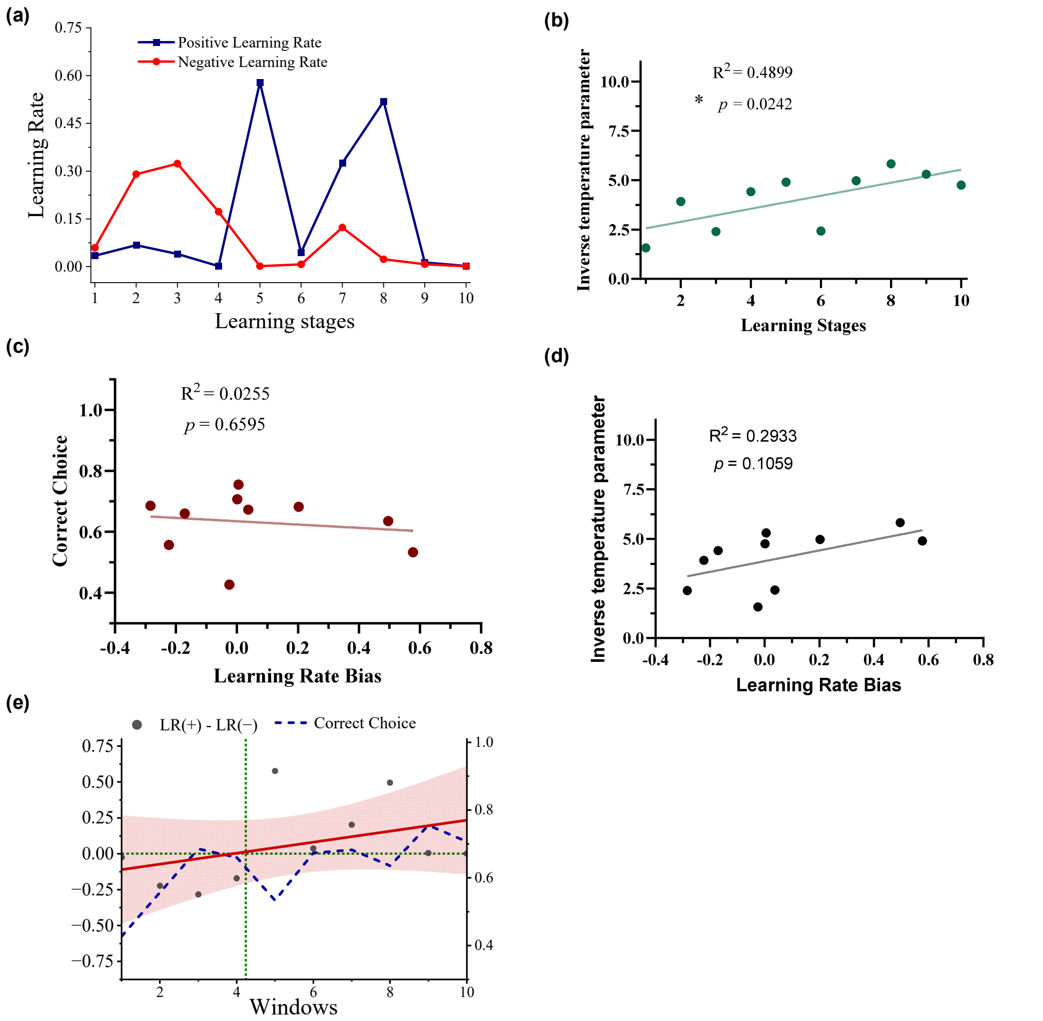

Figure S4. The model results of P029. (a) (a, b) Dynamic learning rate and inverse temperature parameter β of Model 2 (RLB) during the learning process. (c, d) Relationship between learning rate bias and choice performance, inverse temperature parameter β. (e) Dynamic learning rate (LR) bias and choice performance as a function of learning stages.
